# Pre-Operative Oral Health-Related Quality of Life in Patients Attending Surgical Removal of Mandibular Third Molar Teeth

**DOI:** 10.3390/healthcare9010085

**Published:** 2021-01-16

**Authors:** Itzhak Abramovitz, Evgeny Zakopay, Avraham Zini, Harry Chweidan, Daniel Balakirski, Noam E. Protter, Galit Almoznino

**Affiliations:** 1 Department of Endodontics, Faculty of Dental Medicine, Hadassah School of Dental Medicine, Hebrew University, Jerusalem 91120, Israel; itzhakab@hadassah.org.il; 2 Department of Prosthodontics, Oral and Maxillofacial Center, Medical Corps, Israel Defense Forces, Tel-Hashomer 02149, Israel; evgenyzakopay@gmail.com (E.Z.); harryc100@gmail.com (H.C.); balakirski@mail.ru (D.B.); 3 Department of Community Dentistry, Hadassah School of Dental Medicine, Hebrew University, Jerusalem 91120, Israel; aviz@hadassah.org.il; 4 Chief Dental Surgeon & Head of Forensic Unit, Medical Corps, Israel Defense, Tel-Hashomer 02149, Israel; noamprotter@gmail.com; 5 Head, Big Biomedical Data Research Laboratory, Hadassah School of Dental Medicine, Hebrew University, Jerusalem 91120, Israel; 6 Department of Oral Medicine Sedation & Maxillofacial Imaging, Hadassah School of Dental Medicine, Hebrew University, Jerusalem 91120, Israel

**Keywords:** third molar, extraction, dental caries, plaque index, decayed, missing, filled teeth, oral health-related quality of life, oral health impact profile

## Abstract

The study aimed to measure the pre-operative oral health-related quality of life (OHRQoL) and to identify patient and teeth pathologies associated with worse OHRQoL among patients attending mandibular third molar tooth extraction. Data were collected preoperatively from 199 patients attending surgical removal of their mandibular third molar. To that end, we measured the Oral Health Impact Profile-14 (OHIP-14) and analyzed its association with: (1) demographics; (2) health-related behaviors such as smoking, alcohol consumption, physical activity, and dietary habits; (3) Plaque Index (PI); (4) Decay, Missing, and Filled Teeth (DMFT); and (5) clinical characteristics related to third molar extraction, such as the indication for extraction, tooth angulations, and radiographic pathology. The mean age of the study population was 21.5 ± 3.2 years and the mean OHIP-14 global score was 22.5 ± 8.3. The present study identified patient and teeth profiles that are associated with worse pre-operative OHRQoL in patients attending mandibular third molar extraction. The “vulnerable patient” profile includes poor health-related behaviors, particularly the performance of physical activity less than once a week (*p* = 0.028). The “disturbing teeth” profile includes higher plaque scores (*p* = 0.023) and specific characteristics of the third molar teeth, such as pericoronitis (*p* = 0.027) and radiolucency around third molars in panoramic radiography (*p* < 0.001). These findings support the hypothesis that OHRQoL is a complex phenomenon which is associated with the patient’s health-related behaviors as well as with specific tooth pathologies.

## 1. Introduction

The removal of impacted third molars teeth is one of the most common procedures performed by oral and maxillofacial surgeons [1], with two-thirds of all third molars are removed by the age of 30–40 years [1]. The decision to remove an impacted third molar tooth must take into consideration the potential benefits versus the risks of the treatment. The benefits of third molar surgery include the relief of pain, prevention of caries, periodontal disease, dentigerous cyst formation and external root resorption of the adjacent second molar, facilitation of orthodontic treatment, and orthognathic surgery [2]. Therefore, it is recommended to perform the extraction before the development of pathology and associated symptoms [3]. On the other hand, the clinicians should consider possible risk factors for complications, such as an advanced patient age, poor health, and the potential for damage to adjacent structures [4] such as the causing of nerve damage [5].

Considering the risks versus benefits, it is crucial to assess the burden of the presence of an impacted third molar on the patient’s behavioral aspects. Behavioral aspects include health-related habits as well as subjective oral health indicators such as Oral Health-Related Quality of Life (OHRQoL). OHRQoL has gained particular popularity as a tool that characterizes structural, behavioral, and psychosocial consequences of oral diseases [6,7].

While many studies evaluated the OHRQoL post-surgical removal of third molars [8,9], the preoperative assessment has been less studied. Regarding dental aspects, most studies in the literature did not assess the Decayed, Missing, Filled Teeth (DMFT) scores among patients attending third molar extraction, but instead evaluated caries in the second or third molar [10]. Full mouth plaque index (PI) scores among patients attending third molar extraction is also not well studied, and most studies focused on the assessment of periodontal defects in the adjacent second molar [11] or studied the effect of removing impacted third molars on plaque and gingival indices [12]. It is valuable for the dentist to be aware of the predisposition of the patient to other dental morbidities as well as the impacts on OHRQoL before third molar extraction, to enable taking appropriate preventative and/or therapeutic measures.

To shed light on those subjects, the present study aimed to measure the pre-operative OHRQoL in patients attending mandibular third molar tooth extraction and to identify patient and teeth pathologies associated with worse OHRQoL. To that end, we analyzed the association of OHRQoL with (1) demographics, (2) health-related habits, (3) PI, (4) DMFT, and (5) clinical characteristics related to third molar extraction, such as the indication for extraction, tooth angulations, and radiographic pathology. This will enable us to identify patient-related and tooth-related characteristics that are associated with worse OHRQoL. The data of the current study will enable the discovery of demographic, dental, and behavioral profiles of patients attending third molar tooth extraction. The study hypothesized that the worse OHRQoL will be associated with poor patient health-related behaviors and with clinical parameters that are correlated with higher morbidity among patients attending mandibular third molar extraction.

## 2. Study Population and Methods

### 2.1. Study population 

This study included 200 consecutive subjects who were referred to a central dental clinic by general dental practitioners from several dozen clinics through the district for surgical removal of their mandibular third molar tooth. One subject was excluded from the analysis due to missing data, and therefore the final analysis included 199 subjects. The patients were examined and treated at the Medical Corps Center for medical services, Beer-Sheva, Israel.

### 2.2. Sample size calculation 

Winpepi (Pepi-for-Windows): computer programs for epidemiologists and free software developed by JH Abramson [13] that are available online (http://www.brixtonhealth.com/pepi4windows.html) had been used to conduct sample size calculation and showed that at least 188 subjects are required to provide 85% statistical power to identify a 4-point difference by OHIP total score when α = 0.05, and β= 0.15 with an estimated standard deviation of 9.1, based on our previous publication assessing the OHIP-14 [14].

### 2.3. Ethical issues 

The research complies with the Helsinki Declaration guidelines and the STrengthening the Reporting of Observational Studies in Epidemiology (STROBE) protocol. The study was approved by the Tel Hashomer institutional review board (approval number 1118-2011). All patients signed informed consent to participate in the research before their participation. The patients also filled a surgical consent form, which is signed routinely before extractions of third molars. The questionnaires as well as the clinical and radiographic examinations that are part of the study were performed after signing the informed consent to participate in the research and before signing the surgical consent form. This was done in order not to influence the response to questionnaires by reading the warnings written in the surgical consent form.

### 2.4. Eligibility Criteria

**Inclusion criteria:** Subjects of both sexes, aged between 18 and older.

**Exclusion criteria**: Patients with psychiatric illnesses or those taking medications that affect their mental state (such as antidepressants and benzodiazepines); History or the current status of drugs, medications, and/or alcohol abuse; physical disabilities; systemic diseases: e.g., malignant disease, diabetes, cardiovascular diseases, patients with a known defect in the coagulation system (coagulation factors or platelets), patients taking anticoagulants such as coumadin or adenosine diphosphate inhibitors; patients with a background of immunosuppression (after chemotherapy, radiation or chronic administration of steroids), pregnancy or lactation.

### 2.5. Data Collection

The study was based on questionnaires as well as clinical and radiographic examinations for the patients attending third molar extraction. All clinical and radiographic examinations were performed by a single experienced prosthodontics specialist (author EZ).

**Preoperative questionnaires: demographics and behavioral characteristics.** The participants answered self-administrated questionnaires regarding the following demographic and behavioral characteristics:

**Demographics:** age in years; sex (men/women)*,* years of schooling, birth country (native Israelis/immigrants).

**Health-related habits:** including smoking, alcohol consumption, physical activity, and dietary habits, as we described previously [14].

**Assessment of Oral Health-Related Quality of Life (OHRQoL):** OHRQoL was measured using the validated Hebrew version [15] of the Oral Health Impact Profile-14 (OHIP-14) questionnaire [6,7], using domain and global scores. The OHIP-14 global score for each subject was calculated as the sum of the scores of each of the 14 individual OHIP-14 questions. The OHIP-14 includes seven conceptual domains of OHRQoL: functional limitation (question 1 (Q1) and Q2), physical pain (Q3 and Q4), psychological discomfort (Q5 and Q6), physical disability (Q7 and Q8), psychological disability (Q9 and Q10),social disability (Q11andQ12),and handicap (Q13 and Q14). OHIP-14 domains were calculated by summing the response scores for the two corresponding OHIP questions. This method had been described in detail in our previous publications [14,16,17,18].

**Clinical and radiographic examination:** Dental examinations were performed using the aid of light, a dental mirror, and a UNC-15 (University of North Carolina, Chapel Hill, NC, USA) periodontal probe (Hu-Friedy Manufacturing Co., Chicago, IL, USA). Vertical bilateral bitewings including the molar and premolar areas were also included. The following measurements were included:

***Plaque Index (PI).*** Oral hygiene was assessed by the PI and was calculated as the total number of teeth with a visible plaque on any surface of the tooth [19,20].

***Decayed, Missing, Filled Teeth (DMFT) index***. The DMFT index following the World Health Organization (WHO) criteria was used to measure caries experience, which is expressed as the total number of teeth that are decayed, missing, or filled was also evaluated [21], as we described previously [22].

***Assessment of mandibular third molars***. The indication for the prescribed third molar extraction, as well as previous treatment of these teeth before extraction, were included in the clinical evaluation. Bilateral bitewings and panoramic radiographs were performed on all patients. The panoramic assessment included: the angulations of the third molar tooth, the presence of various radiographic pathologies (such as radiolucency around the mandibular third molar tooth and adjacent tooth decay), and proximity to the inferior alveolar nerve.

### 2.6. Statistical Analysis

Data were analyzed using SPSS software version 25.0. A two-tailed level of statistical significance (α) was set at 5%. Continuous variables are presented as means and standard deviations, and categorical variables are presented as frequencies and percentages. Associations between the OHIP-14 global scores and categorical parameters were examined with an independent t- test or Analysis of variance (ANOVA) and with Spearman’s correlation for numerical parameters. Based on the univariate results significant parameters were selected for multivariate linear regression analysis. Multicollinearity tests were added to the analysis.

## 3. Results

The study included 199 patients. One patient was excluded from the study due to missing data. Table 1 presents the demographic characteristics, health-related habits, and dental status of the study population. The mean age of the study population was 21.5 ± 3.2 years and the age range was 18–43 years. Most patients were males (134, 67.3%), were native Israelis (159, 79.5%), and had a mean of 12.2 ± 1.0 years of education. Regarding the health-related habits: most patients were non-smokers (117,58.8%), consumed alcohol mainly on weekends (115, 59.5%) or on social occasions (74, 37.0%), did not perform a specific diet 196 (98.5%), and performed physical activity more than once a week (117, 58.8%). The mean PI score was 1.7 ± 0.7 and the mean DMFT score was 6.7 ± 5.1 (Table 1).

The clinical and radiological evaluation of the mandibular third molar is presented in Table 2. Most of the patients were referred for extraction due to recurring pericoronitis (127, 63.8%) did not have any earlier treatment of the third molar before extraction (135, 67.8%). The angulations of the third molar were usually vertical (142, 71.3%), and in most patients, no radiographic pathology was evident in the panoramic screening (169, 84.9%) Proximity to the inferior alveolar nerve in panoramic was demonstrated in 54.2% of the cases (Table 3).

The global and domain scores of pre-operative OHIP-14 among the study population are presented in Table 3. The mean global OHIP-14 score was 22.5 ± 8.3.

Table 4 presents the associations of the pre-operative OHIP-14 global score with statistically significant parameters among the study population. The following parameters were positively associated with the OHIP-14 global scores (i.e., a worse OHRQoL): smoking (*p* = 0.043) and smoking pack-years (*p* = 0.014), PI (*p* = 0.019), physical activity of less than once a week (*p* = 0.001), alcohol consumption (*p* = 0.020), being on a diet (*p* = 0.02), indications for extraction (from highest to lowest: pericoronitis, occlusal interference, decay in the third molar, prophylactic and adjacent tooth decay; *p* = 0.009), angulations of the third molar in a panoramic context (from highest to lowest: distoangular, horizontal, vertical, mesioangular; *p* = 0.047), radiographic pathology in a panoramic context (from highest to lowest: radiolucency around the third molar, adjacent tooth decay, none; *p* = 0.023), and the number of pericoronitis episodes (from highest to lowest: 3, 2, 1, 0; *p* = 0.047) (Table 4).

The following parameters were negatively correlated with the OHIP-14 global scores: age (*p* = 0.013), and years of education (*p* = 0.011) (Table 4).

There were no statistically significant associations and correlations between the OHIP-14 global score and the following parameters: sex (*p* = 0.138), birth country (*p* = 0.169), D (*p* = 0.467), M (*p* = 0.361), F (*p* = 0.388), DMFT (*p* = 0.645), and proximity to inferior alveolar nerve in panoramic (*p* = 0.519) (data is not presented in a table).

Following the univariate analyses, we performed a multivariate linear regression analysis of the pre-operative OHIP-14 global score as a dependent variable against all the statistically significant parameters. The parameters which retained a statistically significant positive association with the mean OHIP-14 global score following multivariate analysis are depicted in Table 5 and include: PI (*p* = 0.023), pericoronitis as an indication for extraction (*p* = 0.027), and radiolucency around the third molar in a panoramic context (*p*< 0.001) (Table 5). Physical activity had a statistically significant negative association with the mean OHIP-14 global score (*p* = 0.028) (Table 5).

The following variables were included in the multivariate analysis but did not retain a statistically significant association with the OHIP-14 following multivariate analysis: age (*p* = 0.476), years of schooling (*p* = 0.067), smoking (*p* = 0.212), alcohol consumption (*p* = 0.150), dietary habits (*p* = 0.117), and the number of pericoronitis episodes (*p* = 0.108) (data are not depicted in Table 5).

Multicollinearity tests were performed and ruled out multicollinearity [Tolerance > 0.1 and the Variance inflation factor (VIF < 10)] (Table 5).

## 4. Discussion

The current study aimed to identify specific patient-related and teeth-related characteristics that are associated with worse OHRQoL in patients attending the extraction of mandibular third molars. We identified specific demographic, dental, and behavioral characteristics that were associated with worse OHRQoL that will be discussed below.

In our study, patients in the third molar extraction group were on average in their twenties (mean age: 21.49 ± 3.26) and their age was 18–43 years. Similarly, Kautto et al., found that extractions of third molars started in the late teens and peaked at the age of 23–25 and almost two-thirds of the extractions were performed between the ages of 20–39 [23]. Other studies also reported that most third molars were extracted at a mean age of 25 years [24], at 17 or 18 years [25], 29 to 36 years [26], or between 15 and 34 years of age [27].

In the present study, we assessed the pre-operative OHRQoL in patients attending third molar tooth extraction. OHRQoL assesses oral health affecting the physical and social well-being [28]. Higher OHIP-14 scores reflect the negative physical, psychological, and social impact of the presence of third molars. Interestingly, a specific Health-Related Quality of Life (HRQOL) instrument was developed by Shugars et al. to assess recovery following third molar surgery [29]. In the present study, we assessed the preoperative OHRQoL and not post-operative OHRQoL, and therefore we did not use Shugars et al.’s tool. Instead, we used the Slade et al. OHIP-14 instrument, which was developed to be more global, covering a wide variety of oral health conditions and treatments including third molars [6,7]. The OHIP-14 also includes dimensions not addressed by the Shugars et al. condition-specific HRQOL instrument, such as psychological discomfort, psychological disability, and social disability, and these dimensions have been previously shown to be affected by third molar surgery in the short-term [30].

Most published studies evaluated the OHIP-14 post-operatively following extraction [31]. However, in line with our findings of baseline global OHIP-14 scores of 22.5 ± 8.3, Passarelli et al. reported that the mean baseline OHIP-14 scores of patients who underwent third molar surgery were 19.7 ± 9.9 [32]. In the present study, worse OHIP-14 scores were positively associated with worse health-related behaviors such as performing physical activity less than once a week, higher plaque scores, and specific characteristics of the third molar teeth, such as pericoronitis and radiolucency around third molars.

Poor health-related behaviors were associated with a worse OHIP-14 global score, as was previously demonstrated [14]. In particular, a physical activity more than once a week retained a statistically significant association with the OHRQoL in the multivariate analysis. In line with our findings, prior research demonstrated that higher levels of physical activity are associated with better general health-related quality of life (HRQoL) [33,34,35], which was attributed to the physical benefits, biological mechanisms such as elevated endorphin levels, psychological benefits, social interactions, and improvements in self-esteem [36,37]. While physical activity was studied in the context of HRQoL, not many studies addressed the influence of physical activity on Oral HrQoL. We have previously demonstrated a positive association between regular physical activity and better ORHRQoL [14].

In the present study, PI and third molar tooth characteristics but not DMFT scores were positively associated with worse ORRQoL in patients attending third molar extraction. The presence of an impacted third molar tooth may increase plaque, probably due to difficulty in maintaining good oral hygiene [38], which may explain our findings regarding the association of worse OHRQoL with the PI. Third molar pathology and pericoronitis may cause pain and discomfort and interfere with eating, and this may explain their association with worse OHRQoL. Indeed, previous studies demonstrated that impacted third molars may increase a predisposition towards pericoronitis, orofacial infections, dental caries, as well as periodontitis [39,40,41]. In line with our findings, previous studies also demonstrated that clinically important correlations existed between pericoronitis pain and lifestyle and oral function, associations which are not often considered by clinicians or policy makers [42].

## 5. Strengths and Limitations

The main strength of the present study was the relatively large sample size and the homogeneous strict protocol utilizing internationally accepted scores such as PI, DMFT, and OHIP-14, which offer an opportunity to compare our results to other populations and settings. Limitations of the study include the convenience sample, which may limit the generalizability of our results. Due to the case-control study design, we cannot assume causality, and therefore we can only discuss associations and correlations between the variables.

## 6. Conclusions

The present study identified patient and teeth profiles that are associated with worse pre-operative OHRQoL in patients attending mandibular third molar extraction. The “vulnerable patient” profile includes poor health-related behaviors, particularly the performance of physical activity less than once a week. The “disturbing teeth” profile includes higher plaque scores and specific characteristics of the third molar teeth, such as pericoronitis and radiolucency around third molars. These findings highlight the complex phenomenon of OHRQoL, which is influenced by patient’s health-related behaviors as well as specific tooth pathologies. Future studies should further assess the complex phenomenon of OHRQoL in patients attending third molar extraction in other settings and locations, including more covariants and follow-up of patients.

## Figures and Tables

**Table 1:** Demographics, health-related habits, and dental status of the study population.

	Parameter	Mean ± SD	Range
Demographics	Age	21.5 ± 3.2	18–43
	Years of education	12.2 ± 1.0	10–18
	Smoking pack-years	1.6 ± 3.09	0–18
	Parameter	Variable	N (%)
	Sex	Female	65 (32.7)
		Male	134 (67.3)
	Birth country	Native Israelis	159 (79.5)
		Immigrants	40 (20.5)
Health related habits	Smoking	Yes	82 (41.2)
		No	117 (58.8)
	Alcohol consumption	Social occasions	74 (37.0)
		Weekends	115 (59.5)
		Every day	2 (1)
		Never	5 (2.5)
	Dietary habits	Undergoing diet	3 (1.5)
		No specific diet	196 (98.5)
	Physical activity	> Once a week	117 (58.8)
		≤ Once a week	82 (41.2)
Dental status	**Parameter**	**Variable**	**N (%)**
	PI categories	0	6 (3.1%)
		1	76 (39.6%)
		2	79 (41.1%)
		3	31 (16.1%)
	**Parameter**	**Variable**	**Mean ± SD**
	PI score	Score	1.7 ± 0.7
	DMFT	Decayed teeth	2.2 ± 2.5
		Missing teeth	0.5 ± 1.3
		Filled teeth	3.9 ± 3.9
		DMFT	6.7 ± 5.1

**Table 2:** Clinical and radiological evaluation of the 3rd molar extraction group.

Clinical Parameter	Value	N	%
Indication for extraction	Pericoronitis	127	63.8
	Prophylactic	25	12.6
	Decay in the third molar	24	12.1
	Occlusal interference	19	9.5
	Adjacent tooth decay	4	2.0
Previous treatment in the third molar	None	135	67.8
	Antibiotic treatment	60	30.1
	Chlorhexidine mouthwash	4	2.1
Angulations of the third molar in panoramic	Vertical	142	71.3
	Mesioangular	44	22.1
	Horizontal	10	5.0
	Distoangular	3	1.5
Radiographic pathology in panoramic	None	169	84.9
	Adjacent tooth decay	23	11.5
	Radiolucency around 3rd molar	7	3.5
Proximity to inferior alveolar nerve in panoramic	Yes	108	54.2
	No	91	45.7
Number of pericoronitis episodes	0	72	36.0
	1	19	9.5
	2	51	25.5
	3 or more	58	29.0

**Table 3:** Global and domain scores of pre-operative Oral Health Impact Profile-14 (OHIP-14) among the study population.

	OHIP Domain	Mean ± SD OHIP-14 Score
Individual OHIP-14 questions	OHIP question 1	1.1 ± 0.5
	OHIP question 2	1.1 ± 0.4
	OHIP question 3	1.8 ± 1.0
	OHIP question 4	2.4 ± 1.2
	OHIP question 5	1.9 ± 1.1
	OHIP question 6	1.8 ± 1.1
	OHIP question 7	1.5 ± 0.9
	OHIP question 8	1.7 ± 1.0
	OHIP question 9	1.7 ± 1.0
	OHIP question 10	1.4 ± 0.9
	OHIP question 11	1.4 ± 0.8
	OHIP question 12	1.7 ± 0.9
	OHIP question 13	1.3 ± 0.7
	OHIP question 14	1.4 ± 0.8
OHIP-14 domain scores	OHIP 1&2 Functional limitation	2.3 ± 0.7
	OHIP 3&4 Physical pain	4.2 ± 2.0
	OHIP 5&6 Psychological discomfort	3.7 ± 2.0
	OHIP 7&8 Physical disability	3.2 ± 1.7
	OHIP 9&10 Psychological disability	3.1 ± 1.6
	OHIP 11&12 Social disability	3.2 ± 1.6
	OHIP 13&14 Handicap	2.8 ± 1.3
OHIP global score		22.5 ± 8.3

**Table 4:** Associations of pre-operative OHIP-14 global score with statistically significant parameters among the study population (* independent *t* test, ** ANOVA, ˄ Spearman’s correlation).

Parameter	Variable	Mean OHIP-14 ± SD Global Score	*p*-Value
Smoking	Yes	24.0 ± 9.4	0.043 *
	No	21.6 ± 7.4	
Physical activity	≤Once a week	24.5 ± 9.9	0.001 *
	>Once a week	20.7 ± 6.3	
Alcohol consumption	Every day	32.5 ± 21.9	0.02 **
	Never	32.2 ± 9.2	
	Weekends	22.1 ± 8.1	
	Socially consumption	22.4 ± 7.9	
Dietary habits	Undergoing diet	32.0 ± 8.5	0.04 **
	No specific diet	22.1 ± 8.1	
Indication for extraction	Pericoronitis	24.0 ± 9.1	0.009 **
	Occlusal interference	22.3 ± 7.4	
	Decay in the third molar	19.7 ± 5.5	
	Prophylactic	18.8 ± 5.6	
	Adjacent tooth decay	17.0 ± 1.1	
Angulations of 3rd molar in panoramic	Distoangular	28.3 ± 17.2	0.047 **
	Horizontal	25.2 ± 9.6	
	Vertical	23.1 ± 8.6	
	Mesioangular	19.7 ± 6.1	
Radiographic pathology in panoramic	Radiolucency around third molar	29.8 ± 16.7	0.023 **
	Adjacent tooth decay	22.7 ± 7.9	
	None	19.0 ± 6.3	
Number of pericoronitis episodes	0	20.5 ± 6.6	0.047 **
	1	22.7 ± 7.2	
	2	24.3 ± 9.9	
	3 or more	25.7 ± 9.1	
Parameter		Spearman’s rho	*p* value ˄
Age		–0.187	0.013
Years of education		–0.182	0.011
Smoking pack years		0.177	0.014
PI		0.17	0.019

**Table 5:** Multivariate linear regression analysis of OHIP-14 global score as a dependent variable.

	Unstandardized Coefficients		Standardized Coefficients	*p*-Value	95.0% Confidence Interval for B		Collinearity Statistics	
	B	Std. Error	Beta		Lower Bound	Upper Bound	Tolerance	Variance Inflation Factor (VIF)
(Constant)	58.2	12.8		<0.001	32.8	83.5		
Plaque index	1.7	0.7	0.17	0.023	0.25	3.3	0.8	1.1
Physical activity	–2.5	1.1	–0.16	0.028	–4.8	–0.2	0.9	1.0
Indication for extraction = pericoronitis	4.4	2.0	0.27	0.027	0.5	8.4	0.3	3.1
Radiographic pathology in panoramic = radiolucency around third molar	16.2	3.7	0.36	<0.001	8.8	23.6	0.7	1.4

## Data Availability

Data sharing not applicable.
